# Effect of troxerutin in counteracting hyperglycemia-induced VEGF upregulation in endothelial cells: a new option to target early stages of diabetic retinopathy?

**DOI:** 10.3389/fphar.2022.951833

**Published:** 2022-08-15

**Authors:** F. Fahmideh, N. Marchesi, L. I. M. Campagnoli, L. Landini, C. Caramella, A. Barbieri, S. Govoni, A. Pascale

**Affiliations:** ^1^ Department of Drug Sciences, Pharmacology Section, University of Pavia, Pavia, Italy; ^2^ Bausch & Lomb—Iom S.p.A, Vimodrone (Milan), Italy

**Keywords:** troxerutin, VEGF, diabetic retinopathy, hyperglycemia, PKC, ELAV proteins, endothelial cells

## Abstract

Diabetic retinopathy (DR), one of the most common complications of diabetes mellitus, is characterized by degeneration of retinal neurons and neoangiogenesis. Until today, the pharmacological approaches for DR are limited and focused on counteracting the end-stage of this neurodegenerative disease, therefore efforts should be carried out to discover novel pharmacological targets useful to prevent DR development. Hyperglycemia is a major risk factor for endothelial dysfunction and vascular complication, which subsequently may trigger neurodegeneration. We previously demonstrated that, in the rat retina, hyperglycemia activates a new molecular cascade implicating, up-stream, protein kinase C βII (PKC βII), which in turn leads to a higher expression of vascular endothelial growth factor (VEGF), *via* the mRNA-binding Hu-antigen R (HuR) protein. VEGF is a pivotal mediator of neovascularization and a well-known vasopermeability factor. Blocking the increase of VEGF *via* modulation of this cascade can thus represent a new pharmacological option to prevent DR progression. To this aim, proper *in vitro* models are crucial for drug discovery, as they allow to better identify promising effective molecules. Considering that endothelial cells are key elements in DR and that hyperglycemia triggers the PKCβII/HuR/VEGF pathway, we set up two distinct *in vitro* models applying two different stimuli. Namely, human umbilical vein endothelial cells were exposed to phorbol 12-myristate 13-acetate, which mimics diacylglycerol whose synthesis is triggered by diabetic hyperglycemia, while human retinal endothelial cells were treated with high glucose for different times. After selecting the optimal experimental conditions able to determine an increased VEGF production, in search of molecules useful to prevent DR development, we investigated the capability of troxerutin, an antioxidant flavonoid, to counteract not only the rise of VEGF but also the activation of the PKCβII/HuR cascade in both *in vitro* models. The results show the capability of troxerutin to hinder the hyperglycemia-induced increase in VEGF in both models through PKCβII/HuR pathway modulation. Further, these data confirm the key engagement of this cascade as an early event triggered by hyperglycemia to promote VEGF expression. Finally, the present findings also suggest the potential use of troxerutin as a preventive treatment during the early phases of DR.

## Introduction

Diabetic retinopathy (DR) is characterized by degeneration of retinal neurons and neoangiogenesis (Rossino and Casini, 2019). DR is among the leading causes of blindness and it is the principal cause of impaired vision in working-age patients (Leasher et al., 2016; Wong and Sabanayagam, 2020). Moreover, roughly 98% of patients with type 1 diabetes and approximately 78% with type 2 diabetes for more than 15 years will face DR (Nicholson and Schachat, 2010).

Clinically, DR is classified into two stages: non-proliferative diabetic retinopathy (NPDR) and proliferative diabetic retinopathy (PDR). NPDR represents the early stage of DR, wherein increased vascular permeability and capillary occlusion are two main features in the retinal vasculature. PDR, a more advanced stage of DR, is characterized by neovascularization, which is followed by microvascular occlusions leading to progressive retinal ischemia (Wang and Lo, 2018; Semeraro et al., 2019). Although DR has been traditionally considered a retinal microvasculature disease, a neurodegenerative view of the disease has recently emerged. Notably, retinal neurodegeneration is an early process that cannot be reversed. To this regard, hyperglycemia, together with lipid accumulation, is an important amplifier of oxidative stress, which causes dysregulation of cell metabolism and participates in limiting antioxidant defenses during the development of DR. Indeed, diabetes-induced oxidative stress is considered as a key component that dysregulates neurotrophic factors and activates apoptosis, thereby damaging retinal neurons (Ola et al., 2017).

Moreover, the study of DR may have relevance also for other neurodegenerative conditions. Indeed, recent literature data (Wang et al., 2021; Yang et al., 2022) underscore the contribution of vascular cellular elements in neurodegenerative diseases such as Alzheimer’s dementia, making the cellular processes involving them a pivotal element in the study of the mechanisms triggering and sustaining neurodegeneration and in the discovery of new druggable targets. Within this context DR offers a model to study the events taking place at the vascular/neuronal interface (Lynch and Abràmoff, 2017).

Accordingly, these processes are also regulated by numerous mediators, including vascular endothelial growth factor (VEGF, also referred as VEGF-A), which is involved in various events underlying DR progression (Duh et al., 2017). Notably, VEGF is physiologically required for regulating the proliferation and growth of endothelial cells during vasculogenesis. However, several pathological conditions occurring in the course of diabetes upregulate the expression of VEGF (Behl and Kotwani, 2015; Kida et al., 2021), which therefore may lead to increased endothelium permeability, decreased inhibition of pro-apoptotic proteins, activation of various inflammatory mediators, and ultimately neoangiogenesis. Of note, VEGF has a primary role in promoting vascular hyperpermeability, indeed by inducing phosphorylation of endothelial tight junction proteins modulates their degradation, finally fostering blood-retinal barrier disruption (Hicklin and Ellis, 2005; Moran et al., 2016). Moreover, in the retina, hyperglycemia-associated diabetes leads to the generation of diacylglycerol (DAG) that activates protein kinase C (PKC), especially the beta isoform, which is involved in the positive control of VEGF expression (Amadio et al., 2010; Geraldes and King, 2010).

In previous studies, we identified a novel molecular cascade involved in the development of diabetic retinopathy. Specifically, we showed that upon PKCβII activation, the mRNA-binding HuR/ELAV protein is phosphorylated and can bind to VEGF transcript, thus contributing to abnormally enhanced VEGF expression in the retinal tissue (Amadio et al., 2008, 2010). Incidentally, ELAV (embryonic lethal abnormal visual) are RNA-binding proteins (RBP) able to affect the post-synthesis fate of the targeted mRNAs, from the nucleus to the cytoplasm, primarily increasing their cytoplasmic stability and/or translation rate. This family includes HuR, the ubiquitously expressed one, and three neuron-specific members, namely HuB, HuC, and HuD (Pascale and Govoni, 2012; Bronicki and Jasmin, 2013).

Concerning the therapeutic approach, it should be emphasized that treatments for DR are limited and they are mainly focused on the end-stage of this neurodegenerative disease (Duh et al., 2017), therefore efforts should be carried out to discover novel pharmacological targets useful to prevent DR development.

We reasoned that blocking the increase of VEGF through the modulation of the PKCβII/HuR cascade may represent a new pharmacological option to prevent DR progression. This target may also be of interest in other neurodegenerative conditions associated with derangement of the vascular/neuronal interface such as in brain ischemia.

As previously mentioned, hyperglycemia is an important amplifier of oxidative stress, and some studies show that diabetes-induced retinal vascular dysfunction can be indeed prevented by inhibitors of reactive oxygen species (Kowluru et al., 2001). Within this context, it is worth inspecting the beneficial effects of various natural compounds such as flavonoids. As potent antioxidants, flavonoids have been considered useful molecules to protect neurons in the diabetic retina (Al-Dosary et al., 2017; Ola et al., 2017), also by counteracting VEGF production (Chung et al., 2005).

Troxerutin is a flavonoid derived from *Saphora japonica* characterized by a free radical scavenging ability likely responsible for the cytoprotective effect observed in different cell types (Panat et al., 2016).

Troxerutin is a hydroxyethylrutoside; thus, most of its pharmacodynamic and pharmacokinetic properties were evaluated based on this structure (Vinothkumar et al., 2014). There are few direct studies on the pharmacokinetics profile of troxerutin in humans. The oral absorption is not high (it is estimated at approximately 10%). However, it should be noted that the oral absorption of flavonoids is a complex process, and their degree of absorption depends not only on the lipophilicity of the molecules, but also on the influence of transporters and enzymes on the membrane surface (Xin et al., 2018). The maximum reported plasma concentration of hydroxyethylrutosides is 142 μg/L that was reached following a single dose of 900 mg orally administered. This substance has a half-life of 24 h after oral administration and 1 h after intravenous use, suggesting the possibility of once a day oral administration (Aziz et al., 2015). Like other flavonoids, it undergoes hepatic metabolism and is eliminated predominantly *via* the bile system, thus it should be taken with caution in patients with hepatic impairment (Ramelet, 2017). To the best of our knowledge, the maximum daily dose that has ever been used in humans is 7,000 mg orally (Glacet-Bernard et al., 1994). Troxerutin has a safe profile and can cause few adverse reactions, mostly minor gastrointestinal discomfort (Aronson, 2016).

Interestingly, this flavonoid has been shown to reduce neovascularization and VEGF protein production in the retina of diabetic rats (Chung et al., 2005). Moreover, in humans, one study showed that troxerutin given at high doses effectively counteracts retinal vein occlusion thanks to its rheologic properties (Glacet-Bernard et al., 1994).

Therefore, considering that endothelial cells are key elements in DR and that hyperglycemia is a critical factor for diabetes development, we employed two distinct endothelial cell lines applying two different stimuli with the aim of mimicking a hyperglycemia-induced VEGF upregulation *via* activation of PKCβII/HuR cascade. Namely, human umbilical vein endothelial cells (HUVEC) were exposed to PMA (phorbol 12-myristate 13-acetate), which mimics DAG whose synthesis is triggered by diabetic hyperglycemia (Dasevcimen and King, 2007), while human retinal endothelial cells (HREC) were treated with a high glucose concentration for different times.

We then identified the optimal experimental conditions able to determine an increased production of VEGF in both cellular models. Subsequently, in search of molecules useful to prevent DR development, we investigated the capability of troxerutin to counteract not only the rise of VEGF but also the activation of the PKCβII/HuR cascade in both *in vitro* models.

## Materials and methods

### Cell cultures and treatment

HUVEC were obtained from Sigma, plated in 25 cm^2^ flasks, and cultured in an all-in-one ready-to-use medium (Endothelial Cell Growth Medium; Sigma-Aldrich, Milan, Italy). The flasks were incubated at 37°C in a humidified atmosphere containing 5% CO_2_. Cells were treated with PMA at 100 nM and/or troxerutin at different concentrations (see results).

HREC were obtained from Innoprot, plated in fibronectin-coated 75 cm^2^ flasks, and cultured in a specific medium (Endothelial Cell Medium; Innoprot, Bizkaia, Spain) with the addition of 10% Fetal Bovine Serum (FBS), Endothelial Cell Growth Supplement (ECGS), 100 units/mL penicillin and 100 μg/ml streptomycin solution. The flasks were incubated at 37°C in a humidified atmosphere containing 5% CO_2_. Cells were treated with high glucose concentration (25 mM) and/or troxerutin at different concentrations (see results).

### MTT assay

Mitochondrial enzymatic activity was estimated by MTT [3-(4, 5-dimethylthiazol-2-yl)-2,5-diphenyltetrazolium bromide] assay (Sigma-Aldrich). A cell suspension of 3.5 x10^5^ cells/well (for 24 and 48 h) and 5 x10^4^ cells/well (for 72 h and 1 week) in 200 μL was seeded into 96-well plates. Following each treatment, we performed MTT assay following the protocol published in our previous paper (Marchesi et al., 2020). The absorbance values were measured at 595 nm using a Synergy HT microplate reader (BioTek Instruments, Vermont, United States), and the results were expressed as % with respect to control.

### Western blotting

Proteins were measured according to Bradford’s method using bovine albumin as an internal standard. Proteins were diluted in 2x SDS (Sodium Dodecyl Sulphate) protein gel loading solution, boiled for 5 min, and separated onto 12% SDS-PAGE. The anti-HuR mouse monoclonal antibody (Santa Cruz Biotechnology, Santa Cruz, CA, United States), the anti-PKCβII mouse monoclonal antibody (Santa Cruz Biotechnology) and the anti-VEGF rabbit monoclonal antibody (Abcam, Cambridge, MA, United States) were diluted based on each data sheet instructions. Concerning the specific Western blotting procedure, we followed the protocol published in our previous paper (Marchesi et al., 2020). Densitometric analysis were performed using the ImageJ image-processing program.

### ELISA assay for vascular endothelial growth factor

The VEGF protein release into the medium was estimated with the respective ELISA kit (Enzo LifeScience, Farmingdale, NY, United States), according to the relative manufacturer’s instructions.

This test is based on quantitative sandwich enzyme immunoassay technique, where a VEGF specific monoclonal antibody is already pre-coated on a microplate. Standards and samples were pipetted into the wells and any VEGF present was bound by the immobilized antibody. According to the manufacturer’s instructions, we performed a specific number of washes to remove unbound substances. After that, an enzyme-linked polyclonal antibody specific for VEGF was added to the wells. The plate was then washed to remove any unbound antibody-enzyme reagent, a substrate solution was added, and the color developed in proportion to the amount of VEGF bound in the initial step. The color development was stopped with HCl 1N, and the yellow intensity of the color was measured (450 nm) by means of a Synergy HT microplate reader (BioTek Instruments).

### Statistical analysis

The GraphPad Prism statistical package (version 7, San Diego, CA, United States) was used for the statistical analysis. The data were analyzed by analysis of variance (ANOVA) followed, when significant, by an appropriate *post hoc* comparison test, as detailed in the legends. Differences were considered statistically significant when *p*-value ≤ 0.05. The results are expressed as mean ± SD. The N in the legend figure indicates the number of independent experiments, each with 2–3 replicates.

## Results

**FIGURE 1:**
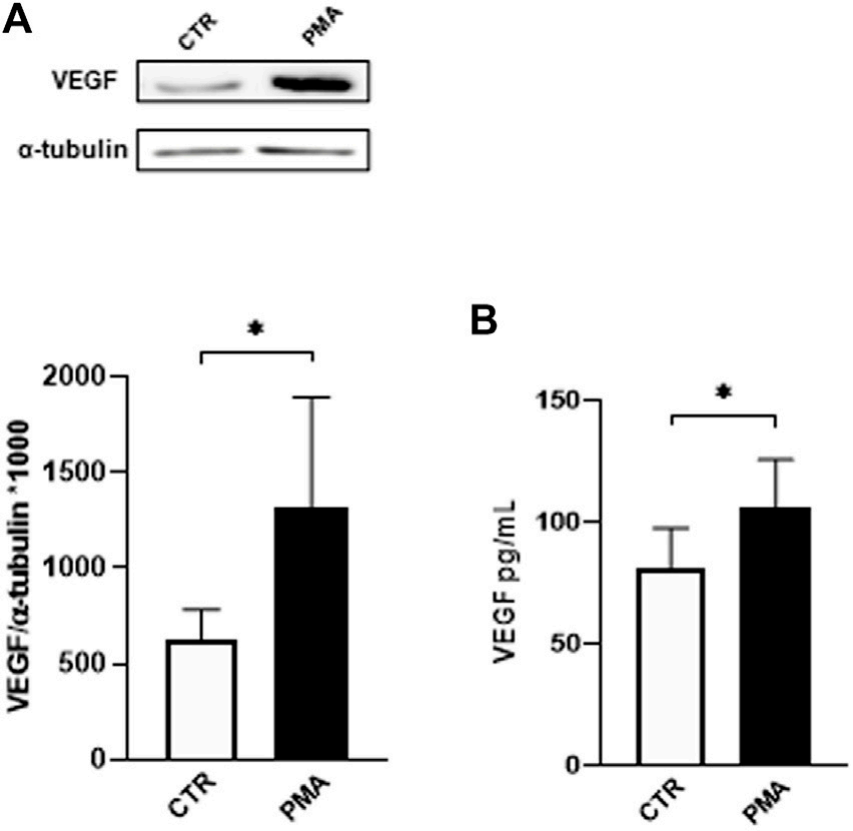
**(A)** Effect of PMA on VEGF intracellular content in HUVEC cells. Cells were exposed to PMA (100 nM) for 48 h. Densitometric analysis of VEGF protein levels. The results are expressed as mean grey levels ratios (mean ± S.D.) of VEGF/α-tubulin immunoreactivities × 1000 measured by Western blotting (upper side: cropped Western blotting images and lower side: densitometric analysis). **(B)** Effect of PMA on VEGF release in the medium of HUVEC cells. Cells were exposed to PMA (100 nM) for 48 h. VEGF protein levels were measured by ELISA. The results are expressed in pg/mL (mean ± S.D.). *p < 0.05, Student’s *t-*test for both intracellular and released VEGF, *n* = 4 independent experiments. CTR, control; PMA, phorbol 12-myristate 13-acetate.

In the first part of the study, we exposed HUVEC to different concentrations of PMA and evaluated the intracellular protein expression of VEGF. The results obtained by Western blotting technique indicate that PMA challenge induces a significant increase in the intracellular content of VEGF following 48 h of exposure at 100 nM (Figure 1A). Using this condition, in parallel, we measured the amount of VEGF released in the HUVEC medium using ELISA technique. As depicted in Figure 1B, HUVEC challenged with 100 nM PMA for 48 h show increased extracellular levels of VEGF.

Subsequently, using a direct *in vitro* approach, we assessed in HUVEC whether troxerutin could affect cell viability. For this purpose, we performed a MTT cytotoxicity assay. The effect of troxerutin addition to the culture medium was tested at different concentrations (10 nM–1 mM) for 24 and 48 h. In the adopted experimental conditions, we found that troxerutin was safe at all the tested concentrations and times of exposure (Supplementary Figure S1). Indeed, a tested product is considered to have a cytotoxic potential only when the cell viability decreases to <70% in comparison to the control group (Srivastava et al., 2018). Considering that, in our experimental conditions, troxerutin was safe up to 1 mM, we performed the following experiments using troxerutin at this concentration. However, to be more confident, we performed additional MTT assays to verify the cell viability of HUVEC exposed for 48 h to more elevated concentrations of troxerutin (10 and 30 mM). The results indicate that even these higher concentrations of troxerutin do not affect the mitochondrial activity of HUVEC cells (data not shown).

In light of the previous findings (see Figure 1), we evaluated the combined effect of PMA (100 nM) and troxerutin (1 mM) exposure on the PKCβII/HuR/VEGF cascade in HUVEC.

**FIGURE 2:**
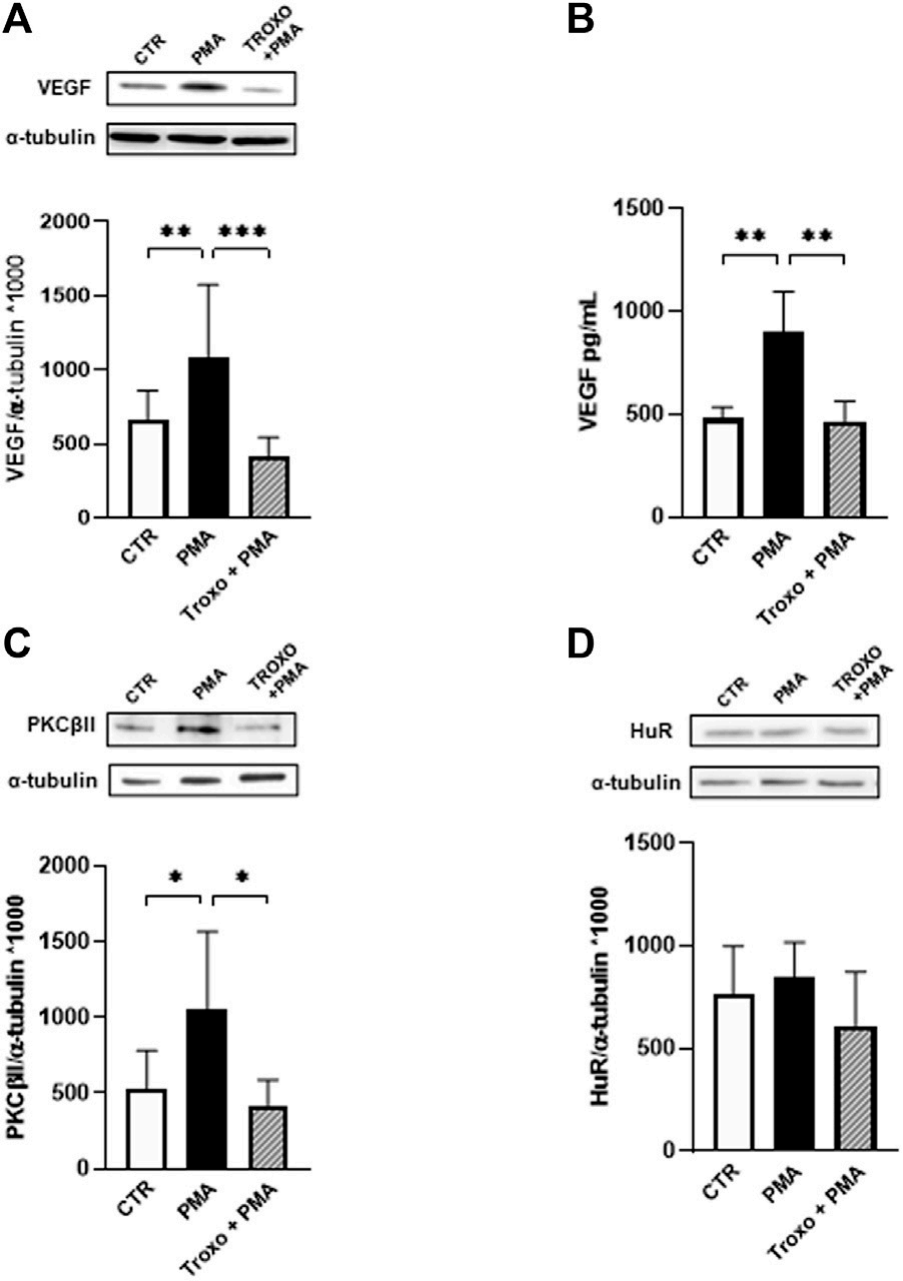
Effect of PMA and troxerutin on VEGF intracellular content **(A)** and on its release in the medium **(B)** in HUVEC. HUVEC were co-exposed to PMA (100 nM) and troxerutin (1 mM) for 48 h. The results are expressed as mean grey levels ratios (mean ± S.D.) of VEGF/α-tubulin immunoreactivities × 1000 measured by Western blotting [**(A)**; upper side: cropped Western blotting images and lower side: densitometric analysis] and VEGF amount in pg/mL (mean ± S.D.) evaluated by ELISA **(B)**. Effect of PMA and troxerutin on PKCβII **(C)** and HuR **(D)** intracellular content in HUVEC. HUVEC were co-exposed to PMA (100 nM) and troxerutin (1 mM) for 48 h. The results are expressed as mean grey levels ratios (mean ± S.D.) of PKC βII/α-tubulin and HuR/α-tubulin immunoreactivities × 1000 measured by Western blotting (upper side a cropped Western blotting image and lower side densitometric analysis). *p < 0.05, **p < 0.01, ***p < 0.001, Dunnett’s Multiple Comparisons test, *n* = 4–8 independent experiments for both intracellular and medium levels. CTR, control; PMA, phorbol 12-myristate 13-acetate; Troxo, troxerutin.

The data, depicted in Figure 2, indicate the capability of troxerutin 1 mM to counteract the increase of both PKCβII and VEGF, the latter both intracellularly (Figure 2A) and in the medium (Figure 2B), induced by PMA treatment. In preliminary experiments, we also explored lower concentrations of troxerutin (10 nM, 100 nM and 1 μM), but none of them was able to hinder the VEGF rise induced by PMA, therefore for the following experiments we used troxerutin at 1 mM. Notably, the mM range is in agreement with the effective concentrations of the compound, found in literature, able to produce an antioxidant action when employing cellular models (Panat et al., 2016).

Concerning HuR protein, we observed a trend, although not significant, towards an increased amount of HuR after PMA exposure that was prevented by troxerutin co-exposure. Nevertheless, since the activation of HuR, through its phosphorylation, is an early event, we assessed changes in HuR phosphorylation status following both PMA and the combined treatment (PMA and troxerutin). After 12 h of PMA administration, we found that HuR phosphorylation was significantly increased at serine residue (*p* < 0.05) and that this rise was prevented by the presence of troxerutin (data not shown).

The second part of the study was conducted on HREC, another relevant cell line within the setting of DR, which are cells isolated from the human healthy retina. We firstly investigated the effect of troxerutin on HREC cell viability/proliferation. Based on the results on HUVEC cells, we decided to focus on the mM range for troxerutin concentrations. Troxerutin’s effect on HREC was tested at different concentrations starting from 1 to 30 mM for 72 h (values % ± S.D.: CTR: 100 ± 4.4; Troxo 1 mM: 103 ± 4.1; Troxo 10 mM: 92.2 ± 4.6; Troxo 30 mM: 71.1 ± 6.4). We observed a slight (around 30%) decrease in cell viability only at 30 mM after 72 h of troxerutin incubation, which is still above the “biocompatibility” threshold. As for HUVEC, we decided to perform the following experiments using troxerutin at 1 mM.

We then investigated the effect of troxerutin on HREC cell viability/proliferation in the presence of continuous high glucose challenge for 72 h and 7 days; to be thorough, we also explored shorter times (24 h, 48 h). The glucose concentration was selected according to Giurdanella et al. work (Giurdanella et al., 2017). The results show no changes in the mitochondrial activity following treatment with glucose (25 mM) at any of the investigated times (Supplementary Figure S2). Further, we also did not observe any change in the mitochondrial activity following the co-incubation of glucose and troxerutin (1 mM) for 72 h and 7 days (data not shown).

**FIGURE 3:**
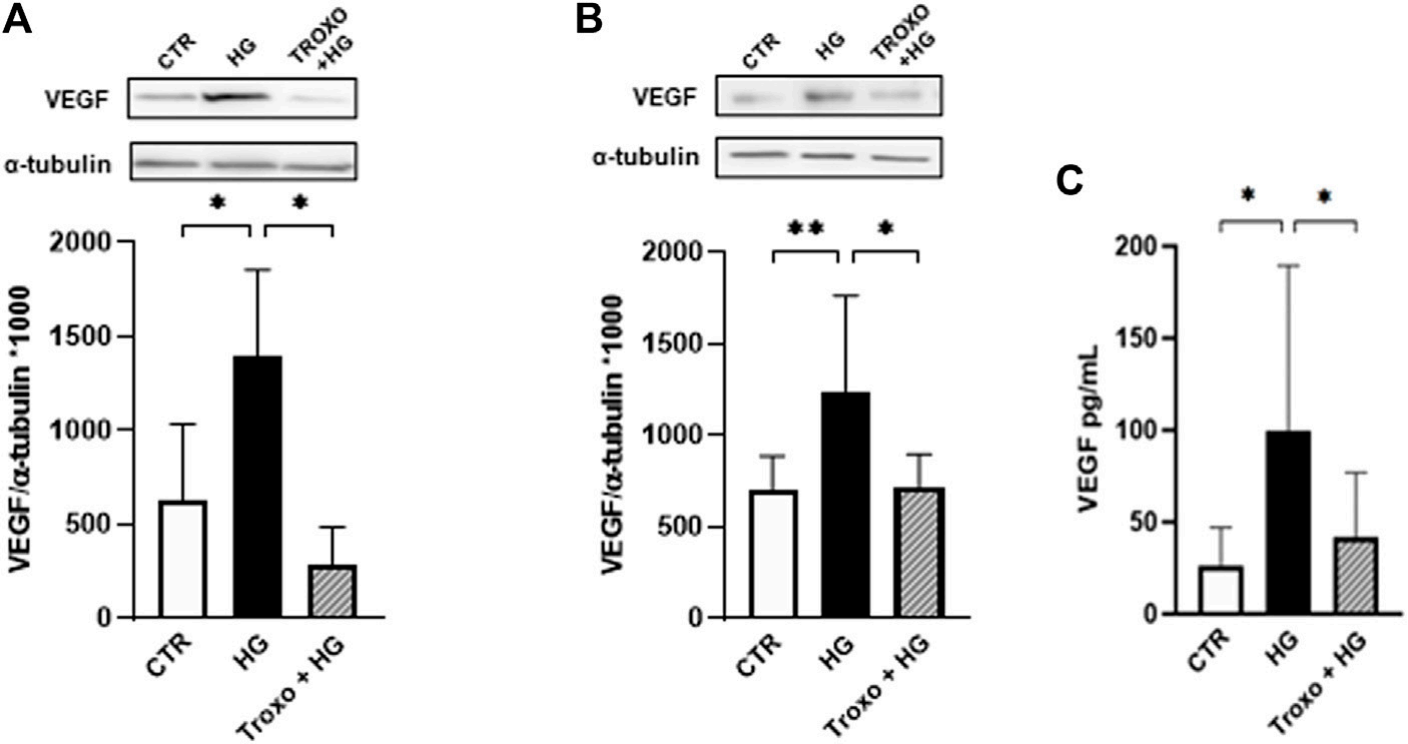
Combined effect of high glucose and troxerutin on VEGF in HREC**.** VEGF was evaluated in the total homogenates of HREC following exposure for 72 h **(A)** and 7 days **(B)** to high glucose levels (25 mM) with or without troxerutin (1 mM). Upper side: cropped representative Western blotting images. Lower side: densitometric analysis. The results are expressed as mean grey levels ratios (mean ± S.D.) of VEGF/α-tubulin immunoreactivities × 1000 measured by Western blotting. **(C)** VEGF release was evaluated by ELISA following 7 days exposure to high glucose levels (25 mM) with or without troxerutin (1 mM). The results are expressed in pg/mL (mean ± S.D.). *p < 0.05, **p < 0.01, Dunnett’s Multiple Comparisons test, *n* = 4–8 independent experiments. CTR, control; HG, high glucose; Troxo, troxerutin.

Thereafter, intracellular protein levels of VEGF were evaluated following 72 h (Figure 3A) and 7 days (Figure 3B) of high glucose (25 mM) exposure with and without troxerutin (1 mM). A significant rise in VEGF protein levels was detected after exposure to high glucose at both times of incubation. Notably, troxerutin 1 mM was able to counteract this increase in VEGF in both the experimental conditions. Moreover, VEGF release was assessed in the medium following 72 h and 7 days of high glucose challenge where we observed a significant increase in VEGF release after 7 days of high glucose exposure. Again, the co-incubation with troxerutin prevented this rise (Figure 3C).

**FIGURE 4:**
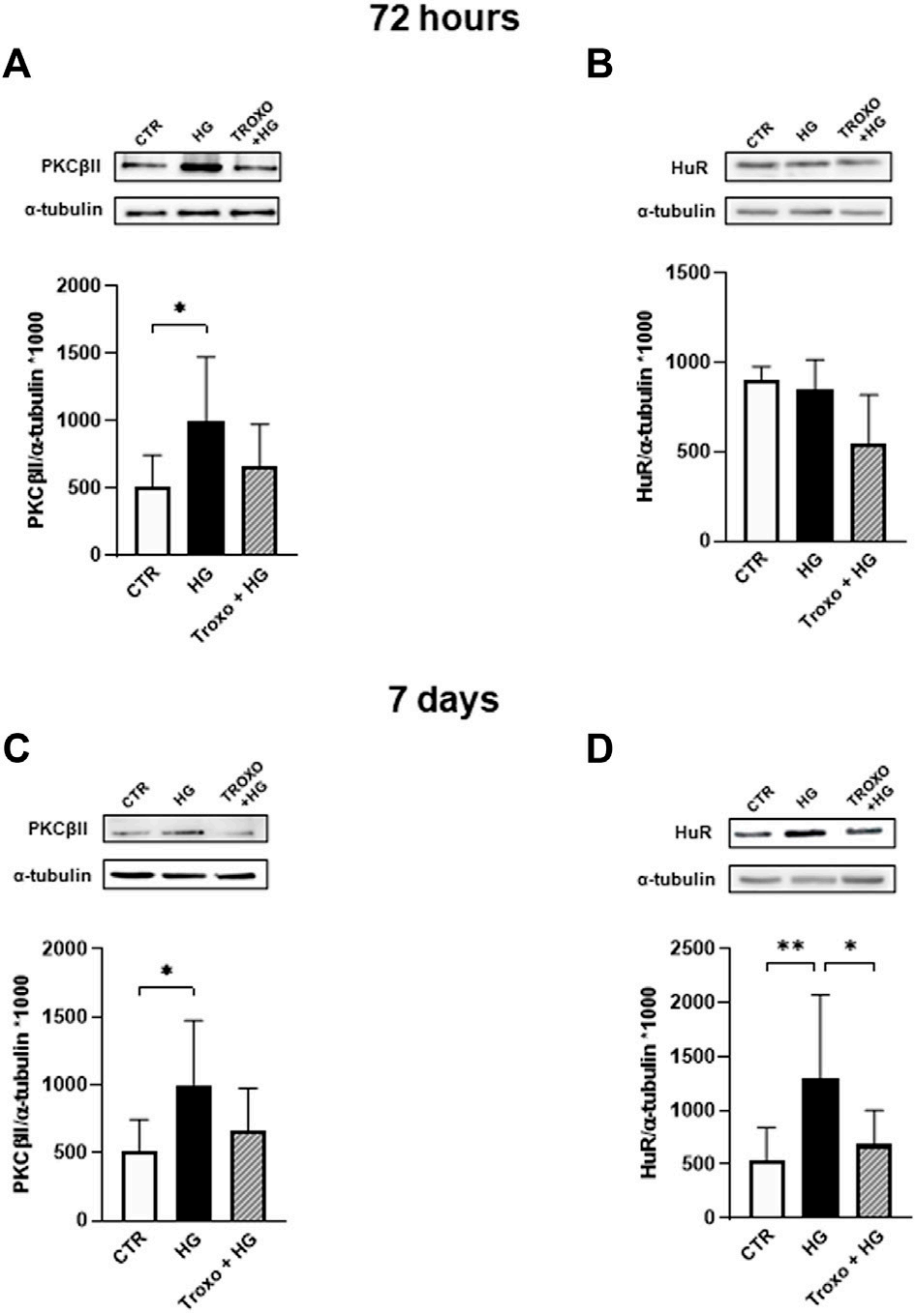
Combined effect of high glucose and troxerutin on PKCβII and HuR in HREC. PKCβII and HuR were evaluated in the total homogenates of HREC following exposure for 72 h **(A,B)** and 7 days **(C,D)** to high glucose levels (25 mM) with or without troxerutin (1 mM). Upper side: cropped Western blotting images. Lower side: densitometric analysis. The results are expressed as mean grey levels ratios (mean ± S.D.) of PKCβII/α-tubulin and HuR/α-tubulin immunoreactivities × 1000 measured by Western blotting. *p < 0.05, **p < 0.01, Dunnett’s Multiple Comparisons test, *n* = 4–8 independent experiments. CTR, control; HG, high glucose; Troxo, troxerutin.

As for HUVEC, we then investigated whether the co-administration of continuous high glucose and troxerutin for 72 h and 7 days was able to affect PKCβII and HuR proteins (Figure 4). Preliminary experiments were also performed at 24 and 48 h (data not shown); however, 72 h and 7 days were the best conditions to further explore the PKCβII/HuR/VEGF pathway. After 72 h of high glucose exposure, we observed a statistically significant increase in PKCβII protein levels (Figure 4A), while no changes were observed in HuR protein (Figure 4B). To this last regard, as previously mentioned for HUVEC, we cannot exclude an activation of HuR itself at this time. Of interest, troxerutin was able to counteract the rise in PKCβII induced by the high glucose stimulus. Following 7 days of continuous glucose exposure, the entire cascade was overexpressed; indeed, we observed an increase in the content of all the examined proteins (Figures 4C,D). Again, troxerutin was able to counteract the rise in the intracellular content of PKCβII/HuR proteins induced by such a prolonged high glucose stimulus.

## Discussion

The study of the events at the interface of vascular/neuronal cells assumes a critical importance in the investigations of DR progression which is sustained by hyperglicemia (Ceriello, 2000; Madsen-Bouterse and Kowluru, 2008; Sun et al., 2010; Cecilia et al., 2019; Meza et al., 2019). Notably, aberrations in endothelial function often precede many of the abnormalities observed in diabetes and may even precede neurodegeneration. To this last regard, it should be underscored that, besides endothelial cells, the neural retina as well could represent an additional target for drug discovery given that the involvement of neuroretina has been found in an *in vivo* model of early DR (Platania et al., 2019).

Although several molecular mechanisms have been invoked to explain the dysfunctions associated with elevated glucose levels, the primary mechanism underlying the endothelial dysfunction in diabetes mellitus remains largely unknown (Maruhashi and Higashi, 2021). Suggested mechanisms include the polyol pathway flux, oxidative stress, and non-enzymatic glycation (Brownlee, 2005). Within this context, a relevant place is undoubtedly taken by PKC, whose activation is related to many vascular abnormalities (Dasevcimen and King, 2007). PKC comprises at least ten serine-threonine kinases that are widely expressed and engaged in a variety of cellular processes (Battaini and Mochlyrosen, 2007; Govoni et al., 2010). It is worth noting that hyperglycemia causes an increase in DAG, the physiological PKC activator. Although other PKC can also be implicated, the PKCβ appears to be the isoenzyme that is largely activated in the retina among the other isoforms (Bucolo et al., 2021). We previously showed that activated PKCβII is able to stimulate the RBP HuR, *via* phosphorylation, which in turn targets VEGF mRNA, finally leading to an increased amount of the correspondent VEGF protein (Amadio et al., 2010; Bucolo et al., 2021).

It should be emphasized that, currently, the most commonly used treatments for DR are mainly aimed at patients who are in the more advanced phases of the disease. Although in many cases these treatments slow the progression of DR and present some effectiveness in preventing vision loss, they are not effective in all patients and are invasive therapies, so new, more compliant therapies that are more effective especially in the early stages of DR are needed (Matos et al., 2020). Therefore, taking into account the lack of compounds useful to prevent the development of this neurodegenerative disease, blocking VEGF upregulation, through the modulation of the PKCβII/HuR pathway, can constitute a novel pharmacological target that can be exploited in drug discovery in the search of effective molecules against DR.

Within this context, even though numerous studies have sought to identify possible treatments for the prevention and treatment of DR, little attention has been given to natural compounds. Indeed, molecules such as flavonoids have been proven to have significant antioxidant and anti-inflammatory effects (Rossino and Casini, 2019). In many animal models and human studies, it has been shown that flavonoids, a large family of compounds that are extracted from plants, can prevent or attenuate complications associated with DR, as they can modulate lipid and carbohydrate metabolism and insulin resistance, mitigate hyperglycemia, suppress oxidative stress and inflammatory processes (Testa et al., 2016).

In this context, we investigated the capability of troxerutin, an antioxidant flavonoid, to affect the PKCβII/HuR/VEGF molecular pathway. In addition to the described antioxidant action (Panat et al., 2016; Al-Dosary et al., 2017; Ola et al., 2017), troxerutin has been also reported to exert several additional pharmacological effects, including anti-inflammatory, antihyperlipidemic, and nephroprotective. Besides, it is suggested to be endowed with some therapeutic roles against neurodegenerative, cardiovascular diseases and diabetes (Zamanian et al., 2020). To this last regard, in a clinical study, the troxerutin-treated group, as compared with the placebo one, showed significant improvement in visual acuity, retinal circulation times, and macular edema; further, treated subjects exhibit diminished progression of ischemia and decreased red blood cell aggregability (Glacet-Bernard et al., 1994). Moreover, in diabetic rats, oral administration of troxerutin at the early stage of DR has been shown to significantly reduce VEGF protein levels compared to controls (Chung et al., 2005).

As mentioned before, we used two different endothelial cell lines challenged with two distinct stimuli: HUVEC with PMA and HREC with glucose. HUVEC cell line provides a classic model system to study many aspects of endothelial functions and disease-associated alterations, such as normal and abnormal angiogenesis, oxidative stress, and inflammation-related pathways. Indeed, HUVEC have been tested to demonstrate stimulation-dependent angiogenesis and key endothelial cell signaling pathways (Kocherova et al., 2019). As a stimulus, we selected PMA, a direct activator of PKC since it mimics its physiologic stimulator, DAG. Indeed, in retinal vascular cells, the early biochemical changes associated with diabetic hyperglycemia leads to the generation of DAG (Dasevcimen and King, 2007). Therefore, PMA is an appropriate stimulus to mimic some of the earliest events induced by high glucose on endothelial cells. Our present data show a significantly increased protein content of VEGF, at both intracellular and extracellular levels, after 48 h of PMA challenge in HUVEC cells. This rise in VEGF seems to rely upon the engagement of the PKCβII/HuR cascade, which acting at post-transcriptional level favors the expression of VEGF. These results confirm our previous findings showing, both *in vitro* (Amadio et al., 2008, 2012; Platania et al., 2020) and *in vivo* (Amadio et al., 2010; Bucolo et al., 2021), the key implication of the RNA binding protein ELAV/HuR in modulating VEGF expression.

Of interest, we document that troxerutin is able to successfully counteract VEGF increase and to hinder, upstream, the activation of the PKCβII/HuR pathway.

A subsequent approach was the use of HREC cells, which are isolated from the human retina and have become a valuable model to examine the effects of diabetes as a whole on the mechanisms of retinal endothelial cell damage and repair (Malek et al., 2018). In this model, we challenged the cells with a physiologic stimulus, glucose, for 72 h and 7 days. Specifically, we observed an increase in VEGF levels, both intracellularly and extracellularly, following the glucose challenge. Once again, the rise in VEGF is mediated by the activation of PKCβII/HuR pathway. Indeed, we observed an upregulation of this cascade, which was more evident following 7 days of glucose exposure. In this last regard and in agreement with our previous work (Amadio et al., 2010; Bucolo et al., 2021), we can hypothesize that HuR, besides promoting VEGF protein expression, can affect not only the expression of PKCβII but also its own expression. Of note, we also document that troxerutin, again, is able to prevent the hyperglycemia-dependent VEGF increase and PKCβII/HuR upregulation.

It should be stressed that troxerutin counteracts the increase of VEGF in both models without suppressing the physiological production of VEGF, which has instead beneficial functions for endothelial cells (Amadio et al., 2016). Indeed, we did not find any significant difference in the amount of VEGF between controls and troxerutin-treated samples (i.e., controls *versus* troxerutin + PMA or troxerutin + high glucose). These results confirm previous data (Amadio et al., 2010) and support the concept that the activation of this cascade is only responsible for the abnormal/detrimental production of VEGF.

**FIGURE 5:**
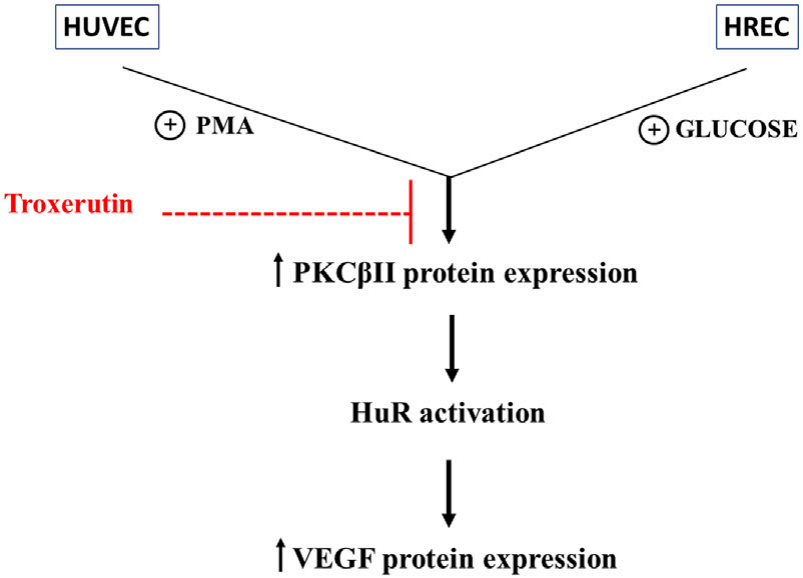
Outline of the effect of troxerutin on the PKCβII/HuR/VEGF cascade activated by hyperglycemia in endothelial cells. The figure shows the engagement of the PKCβII/HuR cascade in determining the increase in VEGF expression within the events triggered by hyperglycemia and indicates the upstream effect of troxerutin in counteracting the upregulation of the entire cascade. We did not investigate in deep the mechanisms of troxerutin-induced reduction in VEGF expression, but it can be supposed that troxerutin may inhibit the over-expression of PKC or its targets in these diabetic *in vitro* models. The analysis of the troxerutin targets through the Swiss TargetPrediction software (http://www.swisstargetprediction.ch) shows that troxerutin may indeed interact with several PKCs, including PKCβII, thus suggesting that additional mechanisms may be studied upstream/downstream its antioxidant actions to better characterize the effect of this substance.

Taken together, the present findings emphasize the engagement of the PKCβII/HuR cascade as an early event triggered by hyperglycemia to promote the expression of VEGF, the primary player in vascular hyperpermeability and endothelial proliferation. Indeed, we confirmed the key involvement of this cascade in two different endothelial cell lines challenged with two distinct stimuli able to directly induce hyperglycemia (high glucose itself) or mimic one of the early consequences of glycemic conditions (the synthesis of DAG) (see Figure 5). Blocking the activation of this pathway can thus constitute a new pharmacological approach to face DR development. Therefore, both models can be helpful within the drug discovery field to assess the potential effect of different compounds as a preventive therapeutic option such as troxerutin. In this regard, we show here that, remarkably, troxerutin is able to preempt the hyperglycemia-induced increase in VEGF in both *in vitro* models, thus suggesting its potential use in DR. It seems that troxerutin ameliorates diabetic retinopathy by downregulating neoangiogenesis factors as well as hindering free radical production, since the latter plays a central role in neuronal degeneration by activating the inflammatory and apoptotic pathways. Indeed, protecting retinal neurons from oxidative stress and hindering VEGF upregulation, together with the consequent vascular damage, through the use of natural substances such as troxerutin may be an efficacious strategy for a preventive treatment during the early phases of DR. However, we cannot exclude the possibility that troxerutin may also act at retinal level also *via* additional mechanisms. For example, a recent work documented that among the gene pathways networks commonly dysregulated in DR retinas are included those linked to fibrosis, another key hallmark of DR that is presently targeted by pharmacological research (Platania et al., 2018). Indeed, troxerutin has been shown to be endowed with an antifibrogenic action (Geetha et al., 2015), therefore future experiments may explore the capability of troxerutin to also counteract retinal fibrosis. Further, considering the implication of genes associated to platelet activation in DR (Platania et al., 2018) and that troxerutin has been reported to improve retinal viscosity microcirculation (Malinska et al., 2019) future studies may also address the effect of troxerutin on platelets function.

Nevertheless, despite all these beneficial effects, troxerutin is manly present into the market in multi-component supplements, hence the present data may direct the market towards the development of troxerutin-based preparations specifically addressed to ocular diseases. Further, future efforts should also be done to explore whether mechanisms as those here described take place at the doses and times that are used in systemic administration in humans, and also following the use of topical formulations. Moreover, it will be of interest to investigate whether the described pathway and troxerutin have a role also in events taking place at the level of cerebral vascular endothelial cells exposed to high glucose concentrations.

## Data Availability

The raw data supporting the conclusions of this article will be made available by the authors, without undue reservation.

## Supplementary material

SUPPLEMENTARY FIGURE S1 Effect of troxerutin on HUVEC mitochondrial activity. HUVEC were exposed to different concentrations of troxerutin for 24 and 48 h. Absorbance values obtained after the MTT assay are expressed as mean percentages ± S.D. over controls (CTR, 100%), *n*=8 independent experiments. CTR, control.

SUPPLEMENTARY FIGURE S2 Effect of glucose exposure on HREC mitochondrial activity. HREC were exposed to high glucose (25 mM) in continuous contact for 24 h, 48 h, 72 h and 7 days. The results are expressed as mean ± S.D. related to MTT measures as a percentage of controls (CTR, 100%), *n*=8 independent experiments. CTR, control; HG, continuous high glucose.
