# Supplementary figures and images for: Effect of troxerutin in counteracting hyperglycemia-induced VEGF upregulation in endothelial cells: a new option to target early stages of diabetic retinopathy?
Source: Front Pharmacol. 2022 Aug 15;13:951833. doi: 10.3389/fphar.2022.951833 (PMC9420903; doi:10.3389/fphar.2022.951833)

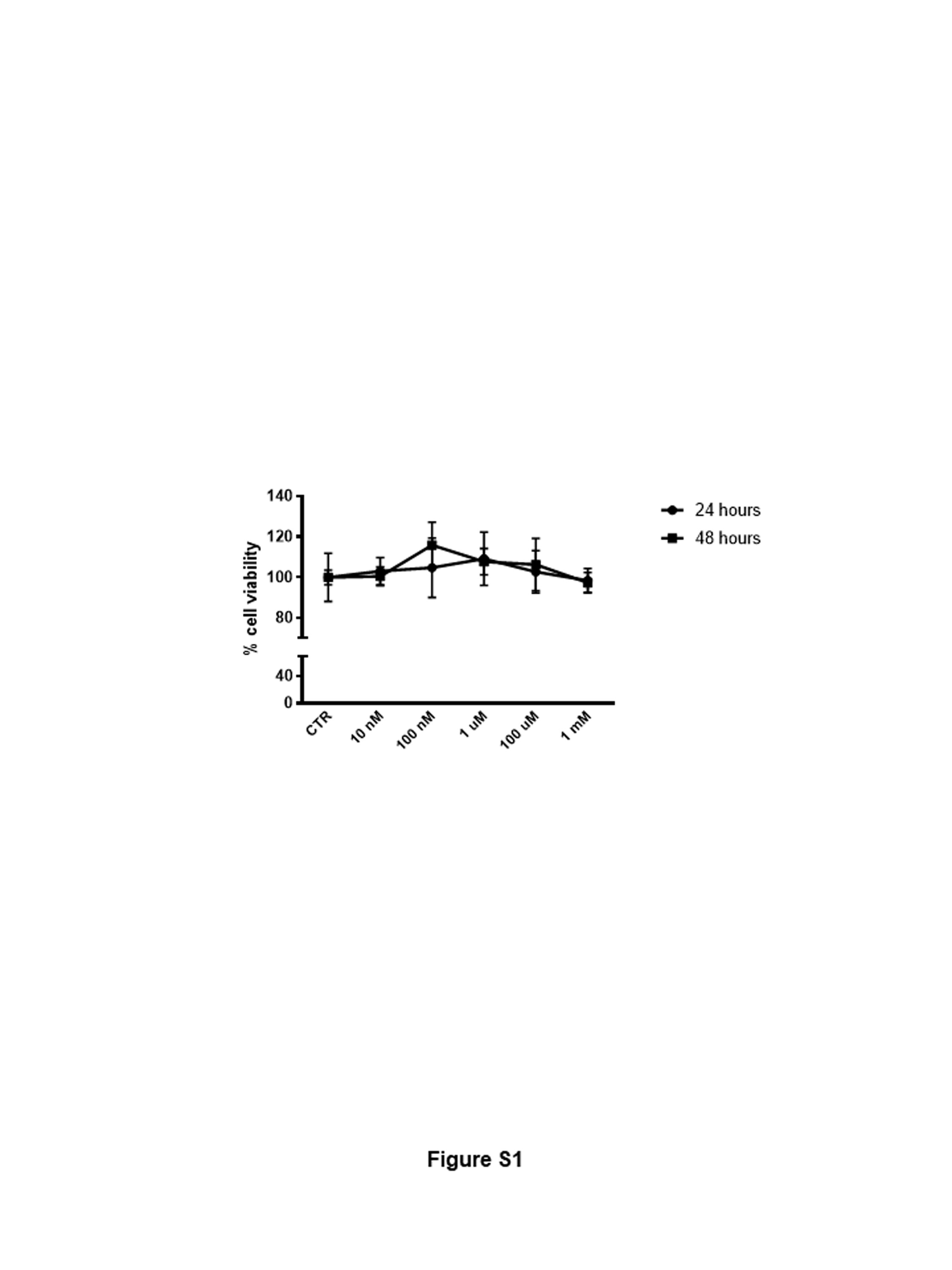

Supplement: Supplementary file 1 [file Image1.JPEG]

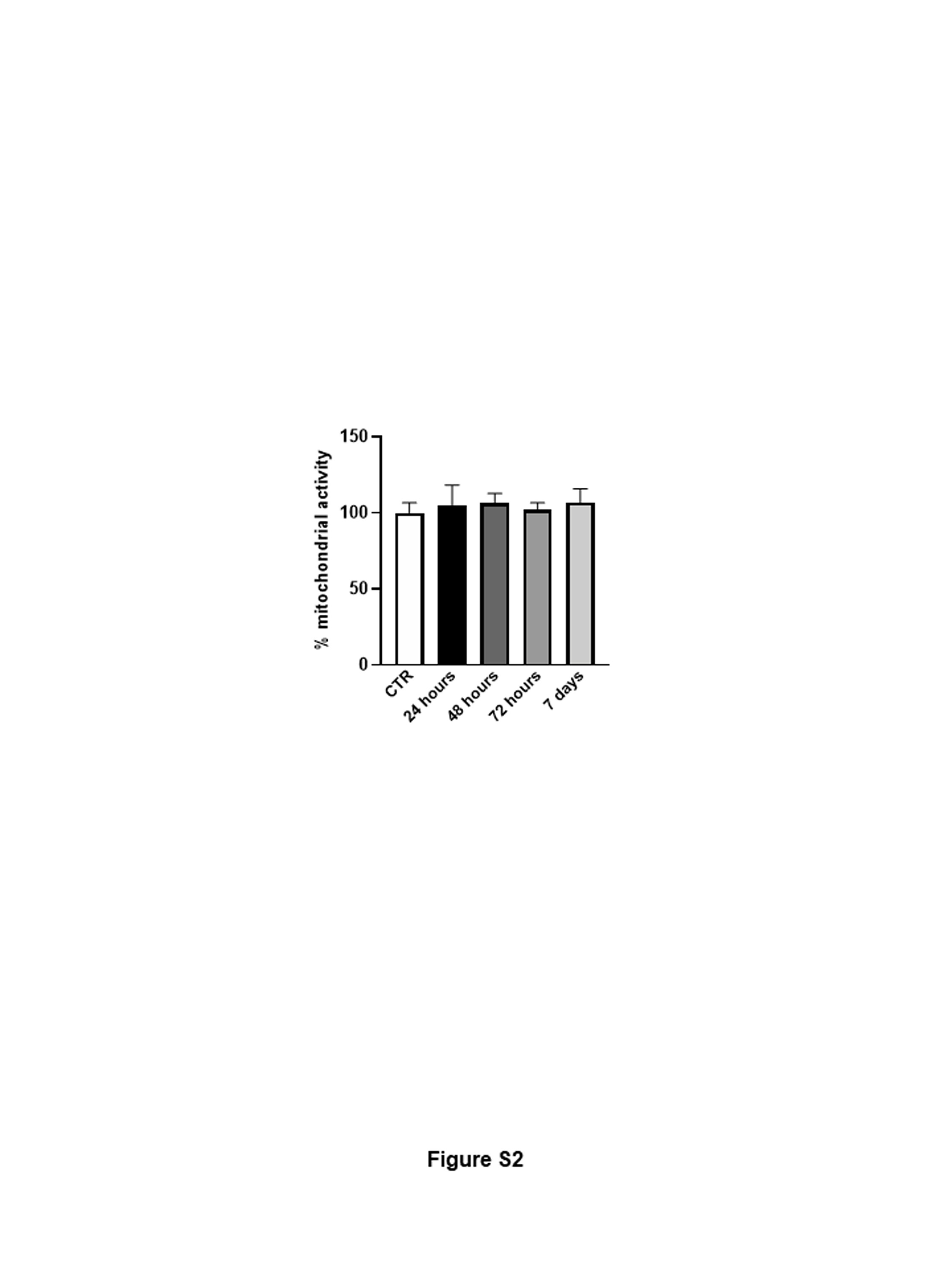

Supplement: Supplementary file 2 [file Image2.JPEG]
